# Chemical Composition, Antioxidant, and Anti-Inflammatory Activity of Essential Oil from Omija (*Schisandra chinensis* (Turcz.) Baill.) Produced by Supercritical Fluid Extraction Using CO_2_

**DOI:** 10.3390/foods10071619

**Published:** 2021-07-13

**Authors:** Jae-Hoon Lee, Yun-Yeol Lee, Jangho Lee, Young-Jin Jang, Hae-Won Jang

**Affiliations:** 1 Korea Food Research Institute, 245 Nongsaengmyeong-ro, Iseo-myeon, Wanju-Gun, Jeollabuk-do 55365, Korea; leejaehoon@kfri.re.kr (J.-H.L.); lyy@kfri.re.kr (Y.-Y.L.); jhlee@kfri.re.kr (J.L.); 2 Major of Food Science & Technology, Seoul Women’s University, Seoul 01797, Korea; 3 Department of Food Science and Biotechnology, Sungshin Women’s University, Seoul 01133, Korea

**Keywords:** omija (*Schisandra chinensis* (Turcz.) Baill.), antioxidant, anti-inflammatory, gas chromatography-mass spectrometry

## Abstract

*Schisandra chinensis* (Turcz.) Baill., which is known as omija in South Korea, is mainly cultivated in East Asia. The present study aimed to investigate the chemical composition of essential oil from the omija (OMEO) fruit obtained by supercritical fluid extraction using CO_2_ and to confirm the antioxidant and anti-inflammatory activity of OMEO using HaCaT human keratinocyte and RAW 264.7 murine macrophages. As a result of the chemical composition analysis of OMEO using gas chromatography-mass spectrometry, a total of 41 compounds were identified. The detailed analysis results are sesquiterpenoids (16), monoterpenoids (14), ketones (4), alcohols (3), aldehydes (2), acids (1), and aromatic hydrocarbons (1). OMEO significantly reduced the increased ROS levels in HaCaT keratinocytes induced by UV-B irradiation (*p* < 0.05). It was confirmed that 5 compounds (α-pinene, camphene, β-myrcene, 2-nonanone, and nerolidol) present in OMEO exhibited inhibitory activity on ROS production. Furthermore, OMEO showed excellent anti-inflammatory activity in RAW 264.7 macrophages induced by lipopolysaccharide. OMEO effectively inhibited NO production (*p* < 0.05) by suppressing the expression of the iNOS protein. Finally, OMEO was investigated for exhibition of anti-inflammatory activity by inhibiting the activation of NF-κB pathway. Taken together, OMEO could be used as a functional food ingredient with excellent antioxidant and anti-inflammatory activity.

## 1. Introduction

Reactive oxygen species (ROS) are known as the representative products of cellular oxygen metabolism [1]. These ROS are regulated to an appropriate level by various enzymes (e.g., catalase, glutathione peroxidase, and superoxide dismutase) present in the body [2]. Otherwise, ROS is overproduced, putting oxidative stress on various tissues, cells, and DNA in the body, eventually causing damage [3]. It is known to cause fatal diseases such as cancer, cardiovascular disease, stroke, and atherosclerosis [1,4]. Reactive nitrogen species (RNS) are another important class of chemically reactive species in the body. Among them, nitric oxide (NO) is a representative RNS, as a small biologically active molecule that plays an important role in various physiological process in the body, such as vasodilation, neurogenesis, sound healing, and modulation of innate immune responses [5]. Therefore, it is important to maintain homeostasis of nitric oxide levels. If homeostasis is broken, it can cause nitrosative damage to biological targets or organelles (e.g., proteins, lipids, DNA, and mitochondria), which can lead to cancer, stroke, or neurodegenerative diseases [6,7]. Nowadays, many scientific researchers are reporting studies to find natural substances with both antioxidant and anti-inflammatory activities that properly regulate ROS and RNS [8,9]. In addition, they have confirmed that there is some relationship between antioxidant and anti-inflammatory activities [8]. This includes the fact that ROS is associated with a variety of inflammatory responses [9,10].

*Schisandra chinensis* (Turcz.) Baill. (Chinese magnolia vine), which also known as omija in South Korea, is mainly cultivated in East Asian countries, including China, Japan, East Russia, and South Korea [11]. This herbal plant belonging to the Magnoliaceae family is well known as a medicinal herb that has long been used to treat diseases such as dyspnea, hepatitis, bronchitis, and coronary heart disease [12,13]. This is due to the various bioactive compounds contained in omija, such as lignans, terpenoids, and polysaccharides [14,15,16]. In particular, according to many studies, lignans are the main bioactive component of omija, and various functional activities, such as antioxidant, anti-cancer, and anti-inflammatory activity, of schisandrin, a representative substance among lignans, have been reported [17,18]. Moreover, in recent studies, there have been reports confirming the antioxidant and immune-regulatory activity of *S. chinensis* fruit extract [19,20]. Hwang et al. [19] reported that *S. chinensis* fruit extract exhibits free-radical-scavenging activity, and Chen et al. [20] reported that *S. chinensis* fruit extract increases the phagocytic activity of macrophages. Through these studies, the possibility of using *S. chinensis* extract as a food additive is suggested, and it is confirmed that it can be widely used as an additive for health promotion in daily products, such as various health food and medicines. Although there are many studies on the functional ingredients of omija, studies on essential oils of omija fruits are insignificant.

Essential oils are secondary metabolites of aromatic plants that are complex mixtures of aromatic compounds, volatile compounds, and hydrophobic compounds from leaves, flowers, and seeds of plants [21]. Various functional effects, such as antioxidant, anti-inflammatory, antimicrobial, antiviral, and anticancer activity, of essential oils extracted from various aromatic plants, including grapefruit [22], *Curcuma caesia* Roxb. [23], and *Zingiberaceae* [24], have been reported. Furthermore, essential oils not only show functional activity, but also have the advantage of reducing various side effects reported by using synthetic compounds [25]. These essential oils have been obtained from aromatic plants in various ways, such as water steam distillation, clevenger hydrodistillation, cold press extraction, and solvent extraction [26]. However, these traditional methods have several disadvantages, like low yields, toxicity of residue solvents, long extraction time, and denaturation or loss of volatile compounds due to heat treatment [27]. Nowadays, the supercritical fluid extraction (SFE) method using carbon dioxide (CO_2_) has begun to be widely used for essential oils extraction and isolation from plants. Compared with traditional extraction methods, SFE has the advantage of increasing extraction efficiency, shortening the extraction time, eliminating the use of harmful organic solvents, and performing extraction at an appropriate temperature so that there is no change in the volatile compounds due to heat treatment [28].

In this study, essential oil of omija (OMEO) was prepared using a SFE method, and its chemical compositions were analyzed using gas chromatography-mass spectrometry (GC-MS). The antioxidant and anti-inflammatory activities of OMEO were determined using HaCaT cells and RAW 264.7 cells.

## 2. Materials and Methods

### 2.1. Materials and Reagents

Dulbecco’s Modified Eagle’s Medium (DMEM), fetal bovine serum (FBS), penicillin-streptomycin, and phosphate-buffered saline (PBS) for cell culture were purchased from Gibco-BRL (Grand Island, NY, USA). Lipopolysaccharide (LPS), thiazolyl blue tetrazolium bromide (MTT), Griess reagent, sodium nitrite solution, the 19 selected volatile compound standards (α-pinene, camphene, β-pinene, sabinene, 3-carene, β-myrcene, α-phellandrene, α-terpinene, limonene, p-cymene, terpinolene, 2-nonanone, acetic acid, citronellal, bornyl acetate, thujopsene, β-chamigrene, (-)-β-bisabolene, and nerolidol) were purchased from Sigma-Aldrich Co. (St. Louis, MO, USA). In addition, 2′,7′-dichlorodihydrofluorescein diacetate (H_2_DCFDA) was obtained from Thermo Fisher Scientific (Carlsbad, CA, USA). Antibodies against iNOS, p65, phospho-p65 (p-p65), and β-actin were obtained from Santa-Cruz Biotechnology (Dallas, TX, USA). Materials for western blot were obtained from Bio-Rad (Hercules, CA, USA), and all other reagents and chemicals used were of analytical grade.

### 2.2. Sample Preparation (Supercritical Fluid Extraction)

Omija fruits were obtained from Hyojongwon Co., a local producer located in Munkyung, Korea. Omija samples were freeze dried and grinded with dry ice using a food-processor grinder (SMX-3500GN, Shinil, Seoul, Korea). The SFE using CO_2_ was performed using a laboratory-scale Supercritical Fluid System (Ilshin Autoclave Co., Daejeon, Korea) to produce OMEO. The extraction conditions were carried out with slight modifications according to Guan et al. [29]. The temperature was 60 °C, the pressure was 350 bar, the extraction time was 2 h, and the CO_2_ flow rate was 40 mL/min. Obtained OMEO were stored at −80 °C in a deep freezer until further analysis. Extract yield of essential of from omija was 6.5%. Extraction yield (% *w*/*w*) was calculated using the following equation:

$$\mathrm{Extract}\;\mathrm{yield}\;\%=\frac{\mathrm{Extract}\;\mathrm{mass}\;\left(g\right)}{\mathrm{Sample}\;\mathrm{mass}\;\left(g\right)}\;\times \;100$$

### 2.3. GC-MS Analysis

The chemical compositions of OMEO were analyzed using an HP 7890 gas chromatograph coupled with an HP 5975 mass selective detector (Agilent Technologies, Palo Alto, CA, USA). All samples were analyzed with a 60 m DB-WAX column (0.25 mm i.d., 0.25 μm thickness, Agilent Technologies). Helium at a constant flow of 1 mL/min was used as the carrier gas. The GC oven temperature programs were as follows: held at 50 °C for 1 min and then increased 3 °C/min to 220 °C. The transfer line, ion source, and quadrupole temperature were maintained at 250, 230, and 150 °C, respectively. The mass spectrum were employed in full scan mode, and the mass range was collected between *m*/*z* 35 and 400. The identification of the chemical compounds of OMEO was carried out using the relative peak area and retention index (Kovat’s index) on DB-WAX. The quantification of chemical compounds contained in 1 g of the sample were relatively quantified using phenethyl alcohol added as an internal standard (ISTD) and the peak area of the identified chemical compounds (ISTD concentration × peak area of each compound/peak area of ISTD) [30].

### 2.4. Cell Culture and Cell Viability

HaCaT human keratinocyte and RAW 264.7 murine macrophages were purchased from the American Type Culture Collection (Rockville, MD, USA) and were cultured in DMEM supplemented with 10% FBS, 1% penicillin/streptomycin at 37 °C in a humidified incubator containing 5% CO_2_.

The effect of OMEO on cell viability was determined using the MTT assay as described by Lee et al. [5]. HaCaT cells and RAW 264.7 cells were transferred to a 96-well plate at a density of 8 × 10^3^ cells/well and 3 × 10^4^ cells/well, respectively. Additionally, each well plate incubated at 37 °C in a 5% CO_2_ incubator for 24 h. After that, OMEO was treated into each well at various concentrations (6.25–100 μg/mL) and incubated for an additional 24 h. MTT (5 mg/mL in PBS) solution was added to each well for an additional 4 h. The supernatant was then removed from each well, and dimethyl sulfoxide was added to each well to dissolve the MTT formazan. The absorbance of each well was measured using a microplate reader (SpectraMax M2e, Molecular Devices, San Jose, CA, USA) at 570 nm. The control used cell medium instead of OMEO. Cell viability was calculated using the following equation:

Cell viability (%) = (absorbance of sample/absorbance of control) × 100

### 2.5. Determination of Intracellular ROS Production

The inhibitory effect of intracellular ROS production of OMEO was measured using H_2_DCFDA fluorescent probe [31]. HaCaT cells (1 × 10^4^ cells/well) were seeded into a 96-well black plate and incubated for 24 h. After then, OMEO at various concentration (6.25–50 μg/mL) were treated for 1 h in the presence of H_2_DCFDA (2 μM). Subsequently, the wells were rinsed three times with PBS and irradiated with 40 mJ/cm^2^ UV-B to generate ROS. The DCF fluorescent were measured using a Fluorescence reader (Molecular Devices) equipped with 485/535 nm excitation/emission filter.

### 2.6. Determination of NO Production

The inhibitory effect of OMEO on NO levels induced by LPS stimulation in RAW 264.7 cells was determined using Griess assay [8]. RAW 264.7 cells (3 × 10^4^ cells/well) were seeded into a 96-well plate and cultured for 24 h. After then, OMEO at various concentration (12.5–100 μg/mL) were treated with LPS (1 μg/mL) for additional 24 h. One hundred μL of supernatant of the medium were added to a new 96-well plate and mixed with 100 μL of the Griess reagent (Sigma-Aldrich). After 15 min incubation at room temperature, absorbance was measured using a microplate reader at 540 nm, and NO concentration was calculated using sodium nitrite standard solution (Sigma-Aldrich).

### 2.7. Western Blot

For western blot analysis [32], RAW 264.7 cells (5 × 10^5^ cells/well) were seeded into a 6-well plate and cultured for 24 h. After then, various concentration (12.5–100 μg/mL) of OMEO were treated with LPS (1 μg/mL) in each well for additional 24 h. Total protein was extracted using RIPA buffer supplemented with protease and phosphatase inhibitor (Thermo Fisher Scientific). The concentration of protein was determined using BCA method. The same concentration (30 μg) of protein was separated on a 8% SDS-PAGE gel and subsequently transferred to polyvinylidene difluoride membranes (Bio-Rad). The membrane was blocked with 5% skim milk for 1 h and then incubated with primary antibody (iNOS, p65, p-p65, and β-actin) overnight at 4 °C. After washing using TBST (Tris-buffered saline with 1% Tween-20) for 45 min (3 times × 15 min), the membrane was incubated with HRP-conjugated secondary antibodies at room temperature for 2 h. After washing using TBST for 45 min, proteins were detected using chemiluminescence reagent (Amersham Pharmacia Biotech, Piscataway, NJ, USA) and observed with G:BOX Chemi XX6 (Syngene Ltd., Frederick, MD, USA).

### 2.8. Statistical Analysis

Results are presented as the mean ± standard deviation from triplicate measurements of the analyses. The Student’s t-test was applied to measure the significance of the difference in mean values between two groups. One-way analysis of variance (ANOVA) followed by Duncan’s post hoc test (*p* < 0.05) was used to measure the significance of the difference in mean values among the multiple groups. All statistical analysis were performed using the SPSS statistics 20 (SPSS Inc., Chicago, IL, USA).

## 3. Results and Discussion

### 3.1. Analysis of Chemical Composition of OMEO

The OMEO obtained by SFE method was analyzed by GC-MS. A total of 41 compounds were identified from OMEO (Table 1 and Supplementary Figure S1), which included sesquiterpenoids (16), monoterpenoids (14), ketones (4), alcohols (3), aldehydes (2), acids (1), and aromatic hydrocarbons (1). The major compounds of OMEO were α-ylangene (3505.49 μg/g), β-himachalene (1163.02 μg/g), (-)-β-elemene (801.15 μg/g), γ-terpinene (473.63 μg/g), and β-chamigrene (472.84 μg/g). These are included in the major groups sesquiterpenoids (75.35%) and monoterpenoids (20.32%), which account for the largest portion of total OMEO compounds.

In our previous study, chemical compounds of omija were analyzed using a headspace stir-bar sorptive extraction-GC-MS [12]. Compared with previous studies, most terpenoid compounds were detected identically, but α-cubebene, α-santalene, thujopsene, α-himachalene, β-chamigrene, and cuparene were newly detected in OMEO obtained by SFE method in the sesquiterpenoids group (Table 1). In addition, bornyl acetate in the monoterpenoids group; 2-hexanone and 2-pentadecanone in the ketones group; (E)-2-heptenal and citronellal in the aldehyde group; and 3-hexanol, 2-hexanol, and nerolidol in the alcohols group were newly detected in OMEO obtained by SFE method (Table 1). The results of this study have also been reported in another previous study using rosemary [33]. In this study, the authors reported that when essential oil was prepared through SFE method, there was a change in chemical composition compared to essential oil prepared through the traditional hydrodistillation methods.

To our best knowledge, this is the first time that the chemical composition of the essential oil extracted from the omija produced by the SFE method via GC-MS has been reported. Some researchers reported the chemical composition of essential oil of omija; however, they analyzed the essential oil obtained through different extraction methods, such as solvent-free microwave extraction [34] and hydrodistillation [35]. Compared with our results, it was confirmed that α-ylangene is the major compound showing the highest content among essential oil compounds. However, in our study, it was found that the proportion of monoterpenoids accounted for 20%, but the content of monoterpenoids in essential oils prepared by other methods was as low as 5% or less [34,35]. As a result, it was confirmed that the chemical composition of the essential oil varies depending on the manufacturing method, which is thought to affect the bio-functionality of the OMEO.

### 3.2. Antioxidant Activity of OMEO in UV-B Irradiation-Induced HaCaT Keratinocyte

HaCaT keratinocyte cells and UV-B irradiation are experimental methods that have been commonly used to determine antioxidant activity. Since UV-B irradiation can induce changes in ROS levels in cells, it is used as a method to measure the ROS scavenging activity of different substances [8,36].

**Figure 1:**
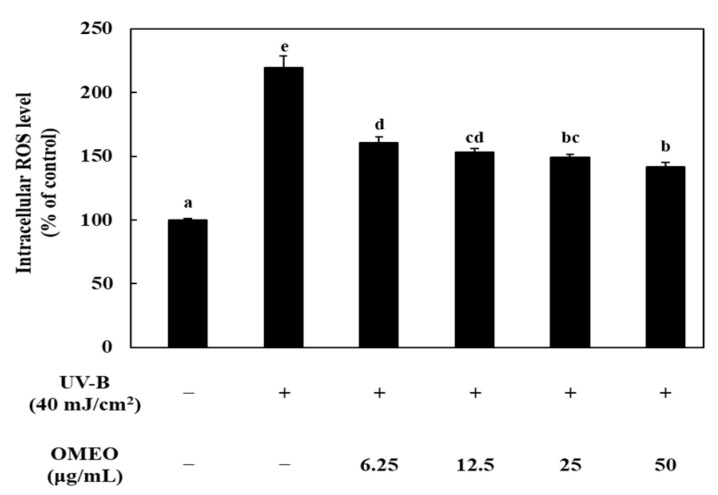
Effect of omija (*Schisandra chinensis* (Turcz.) Baill.) essential oil on the production of ROS in UV-B irradiation-induced HaCaT keratinocytes. Values are expressed as the mean ± standard deviation. Different letters (a–e) among samples indicate significant differences by one-way ANOVA followed by Duncan’s post hoc test (*p* < 0.05). control: group without UV-B irradiation.

Prior to confirming the effects of OMEO on the intracellular ROS levels, the effect on HaCaT cell viability was confirmed by MTT assay. OMEO was not cytotoxic to HaCaT cells below 50 μg/mL. However, from the 100 μg/mL concentration, the cell viability decreased significantly (*p* < 0.05, data not shown). Therefore, the following studies were performed at a concentration of 50 μg/mL or less. The effects of OMEO on intracellular ROS levels are shown in Figure 1. The sample irradiated with UV-B showed increased intracellular ROS, which means that UV-B irradiation acts as trigger to oxidative stress. Compared to the control (non UV-B treated sample), the ROS level of the UV-B irradiated sample significantly increased by about 2.19 fold (*p* < 0.05), and the ROS level was 219.34 ± 9.56%. However, pre-incubation of cells with OMEO resulted in a dose-dependent decrease in ROS levels (*p* < 0.05). At a concentration of 50 μg/mL of OMEO, ROS level was decreased to 141.85 ± 3.53%, and when treated with 25, 12.5, and 6.25 μg/mL, the ROS level was decreased to 148.96 ± 2.77%, 153.32 ± 2.92%, and 160.58 ± 4.81%, respectively.

**Figure 2:**
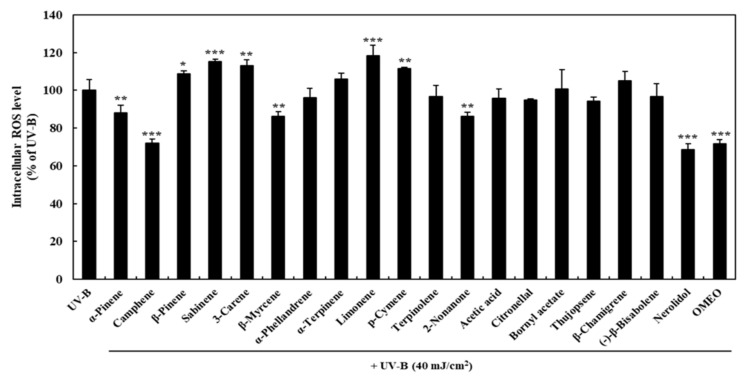
Effects of each compounds (50 μg/mL) of omija (*Schisandra chinensis* (Turcz.) Baill.) essential oil on the production of ROS in UV-B irradiation-induced HaCaT keratinocytes. Values are expressed as the mean ± standard deviation. *, **, *** mean statistical difference for *p* < 0.05, 0.01, 0.001 (Student’s *t*-test) compared with UV-B group.

In order to determine which compound of the OMEO analyzed above is the key compound of OMEO antioxidant activity, the effect of each compound on the ROS level was confirmed. As a result of compound analysis of OMEO, several compounds, including major compounds α-ylangene, β-himachalene, (-)-β-elemene, and γ-terpinene are substances that have already been widely studied for their antioxidant activity [35,37,38]. Therefore, 19 compounds were selected, and the effect on the ROS level was confirmed through the same experiment (19 compounds were purchased from Sigma-Aldrich Co.). The effect of each compound on the ROS level is shown in Figure 2. HaCaT cells were pre-incubated with 50 μg/mL of each compound to determine their effect on ROS levels and compared to the OMEO (50 μg/mL)-treated group. Compared to the UV-B treatment group (100%), the OMEO treatment significantly decreased ROS levels by 71.76 ± 2.22% (*p* < 0.001). Based on the results for each compound, it was confirmed that five compounds (α-pinene, camphene, β-myrcene, 2-nonanone, and nerolidol) significantly decreased the ROS level by 88.03 ± 4.22, 71.93 ± 2.28, 86.27 ± 2.31, 86.11 ± 2.18, and 68.55 ± 3.06%, respectively (α-pinene, β-myrcene, 2-nonanone, *p* < 0.01; camphene, nerolidol, *p* < 0.001). On the other hand, ROS level significantly increased after pre-incubation with five compounds (β-pinene, sabinene, 3-carene, limonene, and p-cymene). However, the ratio of these compounds was less than 1%, so it is thought that there was no significant effect on the ROS-inhibitory activity of OMEO.

Excess intracellular ROS can damage DNA and mitochondria, causing protein oxidation, resulting in abnormal energy metabolism [39]. Moreover, excess ROS can activate the nuclear factor (NF)-κB pathway, and thus, it is known to cause an excessive inflammatory response as well as various diseases, such as cancer, neurologic diseases, and vascular diseases [40]. Therefore, studies on finding antioxidants capable of regulating ROS from various substances are being actively conducted [1,8], and studies on the antioxidant activity of essential oils obtained from various plants have also been reported [37,41]. According to the results of this study, OMEO can reduce the production and accumulation of intracellular ROS content and inhibit the oxidative damage in UV-B-irradiated HaCaT cells. It is suggested that this antioxidant activity originated not only from previously reported antioxidant compounds but also from the α-pinene, camphene, β-myrcene, 2-nonanone, and nerolidol identified in this study.

In particular, overexpression of ROS is known to be closely related to inflammation. Jaisin et al. [36] reported that an increase in intracellular ROS plays an important role in the inflammatory response in human skin. Piperine, an alkaloid compound present in black and white pepper, exhibited strong ROS inhibitory activity in UV-B-irradiated cells and showed anti-inflammatory activity by inhibiting the synthesis of COX-2 and iNOS [36]. Ji et al. [8] reported that peptides from natural plant proteins effectively reduced the production of ROS in H_2_O_2_-induced HaCa T cells and also reported that it has anti-inflammatory activity by inhibiting NO production through the NF-κB pathway in RAW 264.7 cells. Therefore, it is very important to simultaneously confirm antioxidant activity and anti-inflammatory activity.

### 3.3. Anti-Inflammatory Activity of OMEO in LPS-Induced RAW 264.7 Macrophages

The effects of OMEO on NO production in RAW 264.7 cells were measured to determine whether OMEO have anti-inflammatory activity. NO is a small molecule that participates in signaling involved in a wide range of pathophysiological processes, particularly a series of processes related to inflammation [42]. When an inflammatory stimulus occurs, the production of NO increases, which mediates the pro-inflammatory effect. However, overproduction of NO can be harmful and can lead to a variety of inflammatory diseases [43]. Therefore, study of the effect on NO production is being used as a research method to confirm its ability to regulate inflammation.

**Figure 3:**
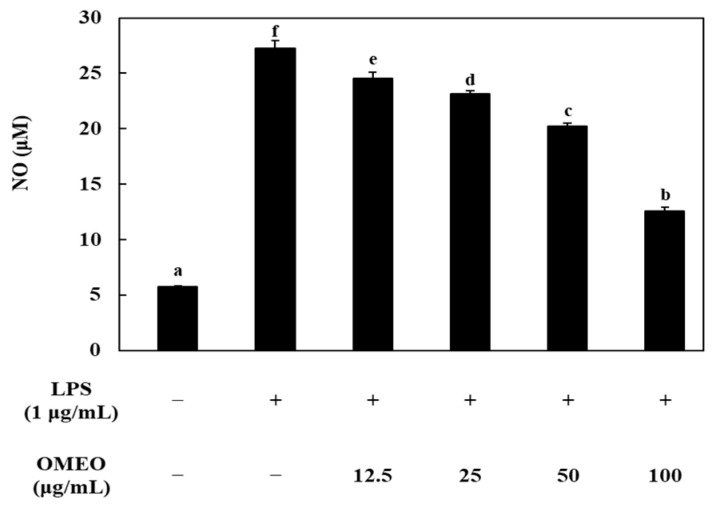
Effect of omija (*Schisandra chinensis* (Turcz.) Baill.) essential oil on the production of NO in LPS-induced RAW 264.7 macrophages. Values are expressed as the mean ± standard deviation. Different letters (a–f) among samples indicate significant differences by one-way ANOVA followed by Duncan’s post hoc test (*p* < 0.05).

**Figure 4:**
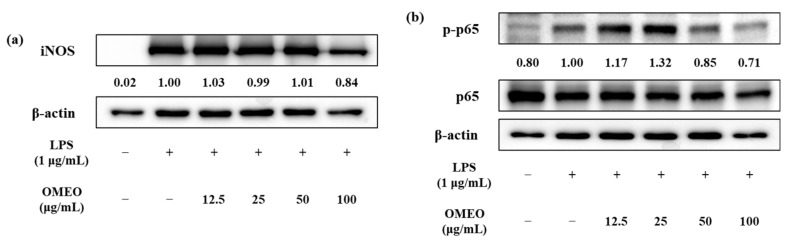
Effect of omija (*Schisandra chinensis* (Turcz.) Baill.) essential oil on the expression of iNOS protein (**a**) and phosphorylation of NF-κB (**b**) in LPS-induced RAW 264.7 macrophages. The relative amounts are expressed as iNOS/β-actin and p-p65/p65, respectively.

Prior to confirming the NO-production inhibitory activity of OMEO, the effect of OMEO on RAW 264.7 cell viability was confirmed by MTT assay. It was confirmed that there was no cytotoxicity at all concentrations of OMEO (12.5–100 μg/mL), and through this, the effect on cytotoxicity was excluded from the NO-inhibition effect (data not shown). The effects of OMEO on NO production are shown in Figure 3. LPS treatment significantly increased NO production compared to the negative control (*p* < 0.05). In the negative control, the amount of NO was 5.73 μM; however, when LPS (1 μg/mL) was treated in RAW 264.7 cells, the amount of NO produced increased by 4.76 times to 27.3 μM. OMEO showed an inhibitory effect on NO production in a dose-dependent manner. At a concentration of 100 μg/mL, OMEO inhibited NO production to 12.6 μM by 52.17%. Additionally, it was confirmed that NO production was suppressed up to 20.3, 23.1, and 24.5 μM by 24.88, 14.61, and 9.69% at concentrations of 50, 25, and 12.5 μg/mL, respectively. This level is a significant decrease compared to the LPS treatment group (*p* < 0.05). NO is synthesized by a family of nitric oxide synthases. In particular, it is known that the synthesis occurs by inducible nitric oxide synthase (iNOS) under stimulation by immunological or microbial stimuli [44]. Therefore, the effect of OMEO on iNOS protein expression was investigated using western blot analysis (Figure 4a). As a result of treating RAW 264.7 cells with LPS, it was confirmed that the expression of iNOS protein was induced compared to the control group (non-treated group). These induced iNOS protein-expression levels were significantly decreased by the treatment with OMEO (100 μg/mL, Figure 4a). As a result, it was confirmed that the inhibitory activity of OMEO against NO production in RAW 264.7 cells was the result of suppressing the expression of iNOS protein.

The pro-inflammatory mediator is mediated by a major transcriptional regulator, such as NF-κB. NF-κB positively mediates the transcription of numerous genes associated with inflammation, such as iNOS, COX-2, chemokines, and inflammatory cytokines [45]. Therefore, studies to find natural products that inhibit the NF-κB signaling pathway are being widely conducted for the purpose of effectively treating various inflammatory diseases [46]. In this study, the effect of OMEO on the phosphorylation of p65, a subunit of NF-κB, was confirmed to determine whether OMEO inhibited NF-κB activation. As shown in Figure 4b, it was confirmed that the phosphorylation of p65 was increased when LPS was treated alone. However, OMEO suppressed the phosphorylation of p65 induced by LPS treatment at 50 and 100 μg/mL concentrations. These results suggest that OMEO inhibits NO and iNOS expression in RAW 264.7 through inhibition of NF-κB activation.

Other scientific researchers have reported that essential oils from various plants have anti-inflammatory activity by inhibiting the production of inflammatory mediators and suppressing the activation of cell signaling pathways [42,46]. These studies also reported that essential oils obtained from *Citrus medica*, *Cirsium japonicum* DC, and *Campomanesia phaea* showed anti-inflammatory activity by inhibiting the NF-κB cell signaling pathway, similar to our results. Therefore, it was confirmed that OMEO have the potential to be used as a safe and effective therapeutic agent for various inflammatory diseases.

In these various studies, research on natural, food-derived substances with antioxidant and anti-inflammatory activity is being actively conducted, and it is reported that these substances can be used as food additives added to functional foods [8,36,42,46]. Therefore, OMEO also has excellent antioxidant and anti-inflammatory activities, as confirmed in this study; it is expected that it can be used in various health functional foods as a food additive in the future.

## 4. Conclusions

In present study, OMEO obtained from *Schisandra chinensis* (Turcz.) Baill. (omija) by supercritical fluid extraction using CO_2_ exhibited antioxidant and anti-inflammatory activities. Through GC-MS analysis, it was confirmed that a total of 41 chemical compounds exist in OMEO. Specifically, OMEO effectively reduced the increased ROS levels in HaCaT keratinocyte cells by UV-B irradiation, which was confirmed to be due to some compounds (α-pinene, camphene, β-myrcene, 2-nonanone, and nerolidol) present in OMEO. Additionally, OMEO significantly inhibited NO production in LPS-induced RAW 264.7 macrophages. It was confirmed that these inhibition effects of OMEO were the result of inhibiting the synthesis of iNOS by suppressing the activation of the NF-κB cell signaling pathway. These findings suggest that OMEO could be used as a functional food ingredient in the food industry in the future. In addition, future research should be accompanied by research on optimization of SFE methods for industrial use of OMEO.

## Figures and Tables

**Table 1:** Chemical composition of omija (*Schisandra chinensis* (Turcz.) Baill.) essential oil.

Peak No.	RT ^1^ (min)	Compound Name	RI ^2^	Concentration (μg/g)	Ratio (%)
1	11.3	α-Pinene	1020	213.19	2.42
2	12.7	Camphene	1065	150.54	1.71
3	13.07	2-Hexanone	1106	92.04	1.04
4	14.17	β-pinene	1108	72.3	0.82
5	14.6	Sabinene	1119	118.69	1.35
6	15.67	3-Carene	1146	18.05	0.20
7	16.05	β-Myrcene	1167	154.8	1.76
8	16.3	α-Phellandrene	1167	32.36	0.37
9	16.88	α-Terpinene	1176	68.01	0.77
10	17.31	3-Hexanol	1199	31.56	0.36
11	17.66	Limonene	1200	88.07	1.00
12	18.1	β-Phellandrene	1212	12.01	0.14
13	18.31	2-Hexanol	1210	53.6	0.61
14	19.61	γ-Terpinene	1243	473.63	5.37
15	20.66	p-Cymene	1266	94.16	1.07
16	21.22	Terpinolene	1280	39.11	0.44
17	22.99	(E)-2-Heptenal	1325	6.82	0.08
18	25.65	2-Nonanone	1386	5.56	0.06
19	27.72	1-Isopropenyl-4-methyl benzene	1421	2.26	0.03
20	28.27	Acetic acid	1461	7.77	0.09
21	28.67	α-Cubebene	1463	4.32	0.05
22	29.45	Citronellal	1474	4.79	0.05
23	29.98	α-Ylangene	1492	3505.49	39.75
24	30.23	α-Copaene	1488	52.88	0.60
25	31.33	β-Bourbonene	1518	10.85	0.12
26	32.01	β-Cubebene	1544	19.41	0.22
27	33.34	α-Santalene	1583	20.2	0.23
28	33.8	Bornyl acetate	1578	255.95	2.90
29	34.07	(-)-β-Elemene	1586	801.15	9.08
30	34.54	β-Caryophyllene	1596	42.76	0.48
31	35.56	Thujopsene	1632	24.29	0.28
32	36.38	α-Himachalene	1656	27.42	0.31
33	37.83	γ-Curcumene	1695	27.79	0.32
34	38.73	β-Chamigrene	1724	472.84	5.36
35	39.17	(-)-β-Bisabolene	1721	282.6	3.20
36	39.9	β-Himachalene	1717	1163.02	13.19
37	40.77	β-Sesquiphellandrene	1771	73.62	0.83
38	42.06	2-Tridecanone	1816	97.77	1.11
39	42.96	Cuparene	1816	117.02	1.33
40	49.19	2-Pentadecanone	2017	15.31	0.17
41	49.68	Nerolidol	2025	64.38	0.73

^1^ RT, retention time ^2^ RI, retention index on DB-WAX.

## Data Availability

Not applicable.

## Supplementary Materials

The following are available online at https://www.mdpi.com/article/10.3390/foods10071619/s1, Figure S1: Chromatographic profile of the *S. chinensis* fruit essential oil by GC-MS.
