# Supplementary material for: Chemical Composition, Antioxidant, and Anti-Inflammatory Activity of Essential Oil from Omija (Schisandra chinensis (Turcz.) Baill.) Produced by Supercritical Fluid Extraction Using CO2
Source: Foods. 2021 Jul 13;10(7):1619. doi: 10.3390/foods10071619 (PMC8304754; doi:10.3390/foods10071619)
Supplement: Supplementary file 1 [file foods-10-01619-s001.zip › foods-1271116-supplementary.pdf]

foods

MDPI

Supplementary Materials

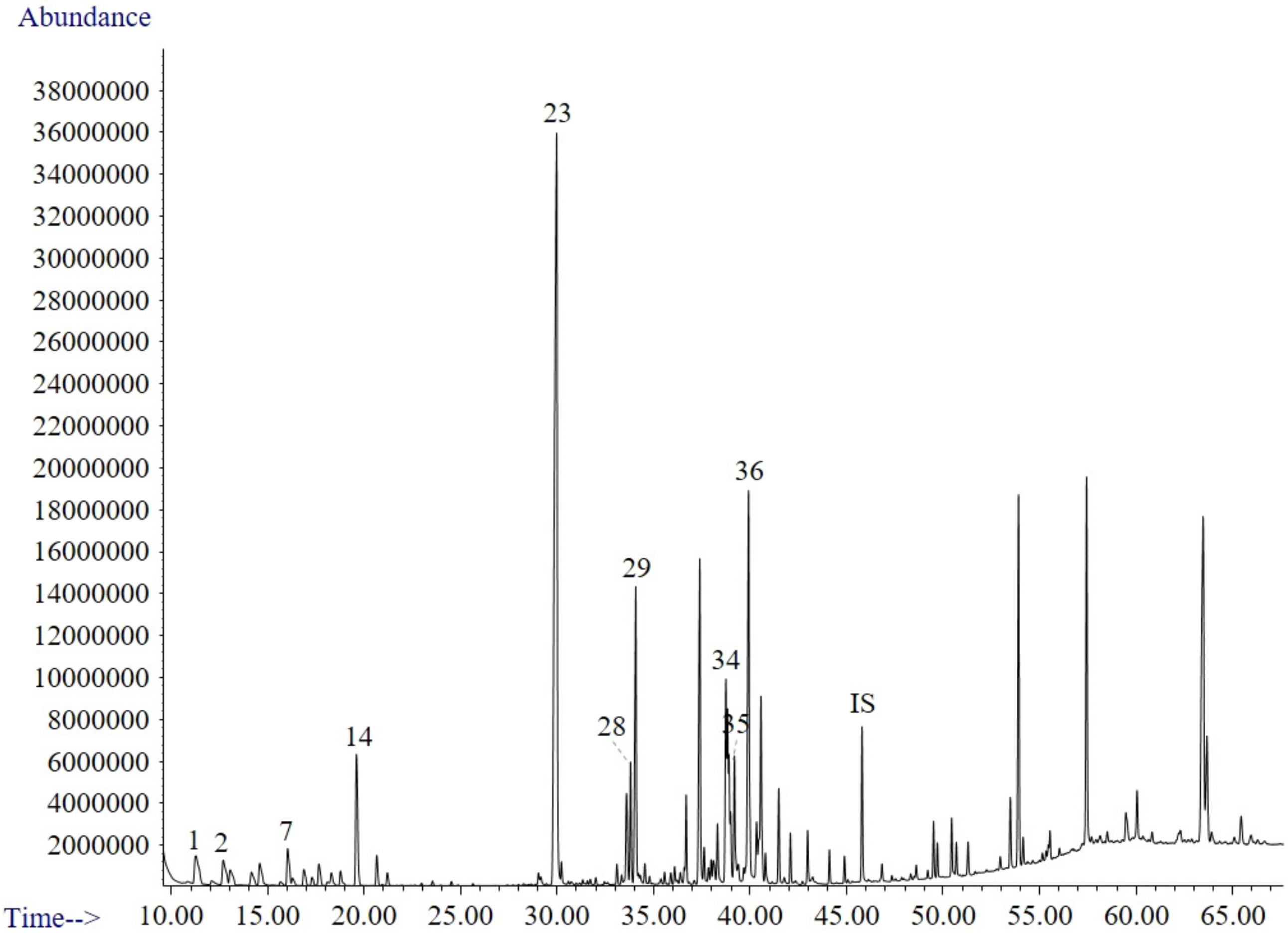

Supplementary Figure S1. Total ion chromatogram (TIC) of the essential oil from *S. chinensis* fruit by GC-MS.

IS: internal standards, 1-36: it is the major compounds and numbered in table 1.
